# News and Notes

**Published:** 1900-03

**Authors:** 


### *NEWS AND NOTES.*

---

### A Variation.

"An inch and a half incision,
A week and a half in bed,"
Then ho! for the fields Elysian!
Peace to the quiet dead.

---

*Record of Symptomatology* is a new publication in Omaha, Neb. The first number has just been issued.

---

Dr. John R. Humphrey, one of the most prominent physicians in his section, died at his home in Acworth, Ga., on Feb. 7th.

---

The *North Carolina Medical Journal* has changed its name to Carolina, since it is now the representative journal of those two States. We wish it success.

---

Dr. J. C. Wilson, of Valdosta, was married on February 6th to Miss Ola Halzendorff of Waycross. Dr. Wilson is a prominent young physician in South Georgia.

---

The medical faculty at Heidelberg now admits women students on the same footing as men, if provided with the diploma of a German "gymnasium" or high school course.

---

A patient recently died in this city who was at that time under the medical and spiritual care of the local Christian Scientists. Physicians refused to sign his death certificate, but finally out of the goodness of his heart the President of the Board of Health, Dr. Jarnagin, arranged the necessary certificate.

CLEVELAND has an Appendicitis Club, to be eligible to which it is necessary to have a surgeon's certificate that the applicant has undergone an operation for that disease. The Chicago *Daily News* suggests that another operation on their brains would be interesting to the general public as showing what quality of gray matter, if any, goes to the formation of the nominal thinking apparatus of the members.

---

MR. J. PIERPONT MORGAN, who some time ago gave $1,000,000 to the Society of the Lying-in Hospital, New York, for the erection of a new hospital building, has added to his donation the sum of $350,000 and two large lots which will give an increased area for building purposes of about four thousand square feet. The new building will, it is understood, be ready for occupancy in about a year.

---

A CHAIR of intertropical pathology has been created at Havana and Dr. Juan Guitéras appointed to it. He is at present studying in Europe. The *Archivos de la Policlinica* congratulates the Havana faculty on "this brilliant and exceptional acquisition," and expresses its exalted appreciation of "the patriotic act of the doctor in leaving his position in Philadelphia—the chair of pathologic anatomy at the University of Pennsylvania—to return to Cuba for our welfare and to work for medical culture."

---

THE fashionable street costume for women now is—'tights.' Still, a large proportion of the women one sees on the street do not seem to be borrowing their notions of decency altogether from the 'demi-monde' of Paris.—*Modern Medical Science.*

The style of skirt now worn might be called the "Aphrodisiac." Whatever be the intention, the effect is more pronounced than a mormon wafer.

A FRENCH journal states that there are no fewer than twenty-five medical men attached to the Russian court. There is, first, a physician-in-chief, then comes the physicians-in-ordinary and the honorary physicians. The surgeons-in-ordinary number three, and there are four honorary surgeons. Of ophthalmologists there are two, and other specialists abound in proportion. The Czarina has a physician, a surgeon, and three specialists. In addition to this goodly array there are a chiropodist-in-ordinary and an honorary chiropodist. The cynical may find here a satisfactory explanation of the alleged wretched health of this poor man.

---

MEDICAL ethics is to be represented at the International Medical Congress as a special department of medical deontology. A circular has been sent to various medical organizations asking them to appoint delegates. The proceedings are to be in English, French, German and Italian. This congress is to take up especially the subjects of medical charity, hospital practice and medical ethics. We would strongly recommend that one of the war-horses of the American Medical Association have a copy of the Code of Ethics and of the discussions of the American Medical Association anent this valuable document bound in flexible covers, and read this to the assembled congress. We would be very much interested to learn what foreigners thought of this valuable American medical product.—*Medical Review.*

---

A BILL has been prepared in Colorado which, if passed, will place the granting of marriage licenses in that State in the hands of a board of medical examiners. In each county there is to be a board, to consist of three physicians, no two of the same school; and where possible the board is to have one or more female members. Licenses are to be granted to men not less than twenty-five years old and to women not less than twenty-two. To secure licenses men and women must be free from dipsomania, insanity, tuberculosis, hereditary asthma, scrofula, epilepsy and other diseases. There must be no blood relation between parties who wish to contract. No certificate entitling one to receive a license shall be granted to a person notorious for moral depravity or to one who shall at the time of the application be on trial, under bonds, or in prison for felony.

---

**This Journal and Atlanta Semi-weekly Journal one year for $1.00 cash in advance. No checks.**

---

SURGEON-GENERAL STERNBERG has prepared a bill for presentation to Congress providing for an increase of men in the Medical Department of the Army. This increase is necessitated by the expansion in the military organization. The bill provides for an addition to the present corps of four assistant surgeon-generals with the rank of colonel; ten deputy surgeon-generals with the rank of lieutenant-colonel; thirty surgeons with the rank of major; and eighty assistant-surgeons with the rank of lieutenant, who will have the rank of captain at the expiration of five years' service. These positions are to be filled by seniority promotion in accordance with the established laws and regulations. Acting assistant-surgeons to the number authorized are to be appointed subject to the usual examination for a probationary period of six months, during which time they will attend the Army Medical School in Washington. At the end of this time if their standing is satisfactory they will be commissioned to fill existing vacancies. —*Medical Courier*.

